# Continuous Quadrupole Magnetic Separation of Islets during Digestion Improves Purified Porcine Islet Viability

**DOI:** 10.1155/2016/6162970

**Published:** 2016-10-23

**Authors:** Bradley P. Weegman, Venkata Sunil Kumar Sajja, Thomas M. Suszynski, Michael D. Rizzari, William E. Scott III, Jennifer P. Kitzmann, Kate R. Mueller, Thomas R. Hanley, David J. Kennedy, Paul W. Todd, Appakalai N. Balamurugan, Bernhard J. Hering, Klearchos K. Papas

**Affiliations:** ^1^CMRR, Department of Radiology, University of Minnesota, Minneapolis, MN, USA; ^2^Schulze Diabetes Institute, Department of Surgery, University of Minnesota, Minneapolis, MN, USA; ^3^Department of Chemical Engineering, Auburn University, Auburn, AL, USA; ^4^Department of Surgery, University of Arizona, Tucson, AZ, USA; ^5^MobileMedTek Inc., Louisville, KY 40206, USA; ^6^Techshot, Inc., Greenville, IN, USA; ^7^Clinical Islet Cell Laboratory, Cardiovascular Innovation Institute, Department of Surgery, University of Louisville, Louisville, KY 40202, USA

## Abstract

Islet transplantation (ITx) is an emerging and promising therapy for patients with uncontrolled type 1 diabetes. The islet isolation and purification processes require exposure to extended cold ischemia, warm-enzymatic digestion, mechanical agitation, and use of damaging chemicals for density gradient separation (DG), all of which reduce viable islet yield. In this paper, we describe initial proof-of-concept studies exploring quadrupole magnetic separation (QMS) of islets as an alternative to DG to reduce exposure to these harsh conditions. Three porcine pancreata were split into two parts, the splenic lobe (SPL) and the combined connecting/duodenal lobes (CDL), for paired digestions and purifications. Islets in the SPL were preferentially labeled using magnetic microparticles (MMPs) that lodge within the islet microvasculature when infused into the pancreas and were continuously separated from the exocrine tissue by QMS during the collection phase of the digestion process. Unlabeled islets from the CDL were purified by conventional DG. Islets purified by QMS exhibited significantly improved viability (measured by oxygen consumption rate per DNA, *p* < 0.03) and better morphology relative to control islets. Islet purification by QMS can reduce the detrimental effects of prolonged exposure to toxic enzymes and density gradient solutions and substantially improve islet viability after isolation.

## 1. Introduction

Pancreatic islet transplantation is emerging as a promising treatment method for select patients with type 1 diabetes [1, 2], but there are many hurdles to overcome before transitioning to larger scale implementation. Using the current islet isolation and purification processes, the quantities of islets needed to achieve insulin independence are much higher than expected; patients often require multiple islet infusions from two or more donors for insulin independence [3–5]. Islet isolation and pancreas allotransplantation have recently approached similar success rates for maintaining insulin independence five years following transplantation. It is important to note that achieving this similarity in 5-year outcomes requires islets from 2-3 donated pancreata as opposed to a single whole pancreas transplant. Some centers have demonstrated success with single donor islet transplants with highly selected donors and recipients, but it is clear that this cannot be achieved routinely [6, 7]. The shortage of suitable donor pancreata, the cost of the procedure, and the need for systemic immunosuppression currently limit the application of this encouraging treatment [6].

Many factors contribute to islet yield and viability including donor related parameters, pancreas procurement and preservation methods [8–12], and especially the islet isolation process itself. Many islets are never retrieved during isolation [13] or are irreversibly damaged along the way [14–16]. The standard isolation processes involve a digestion step to liberate islets from the surrounding parenchyma and a purification step to separate and purify the islets from the digested exocrine tissue. Other alternative methods are being explored [17, 18], but the standard digestion procedure uses strong proteolytic enzymes that are infused into the pancreatic ductal system to preferentially digest exocrine tissue at 37°C. The digestion process is harmful and has been shown to damage islets [19, 20], but the purification step also damages islets most likely synergizing with the stresses encountered throughout the islet isolation process. Islets, making up only about 2% of the total pancreatic mass, are purified after digestion to decrease the volume of tissue being transplanted and to eliminate unnecessary and potentially harmful exocrine tissue. The exocrine tissue itself releases endogenous enzymes that may contribute to islet damage and loss in culture [21]. Coculture of islets with contaminating exocrine tissue has been shown to decrease the overall viability of the preparation, which may have lasting effects decreasing the success of the graft after transplant. Exocrine contamination may also directly affect graft health after transplant by hindering vascularization [22] and eliciting damaging inflammation and immunologic responses [23, 24].

Current islet purification processes rely on a density gradient centrifugation technique to separate the less dense islets from the more dense exocrine tissue. This method exacerbates the damage islets experience during digestion by prolonging exposure to proteolytic enzymes. Furthermore, islets continue to experience substantial stresses throughout the purification process including extended hypoxia, abrupt temperature changes, and mechanical shear stresses, as well as prolonged exposure to reactive oxygen species, hyperosmolar solutions, and proinflammatory cytokines [25–28]. All of these factors contribute to the overall reduction in viability and potency of the islet preparation. In addition, the density purification process involves a collection step, where islets are concentrated into pellets, which creates a hypoxic environment. This is a paramount concern because islets are known to be especially sensitive to warm and cold ischemia due to the lack of ability to cope with hypoxia [29–32].

Following the collection step, the islets are exposed to the density gradient chemicals (commonly Ficoll® or similar) and then centrifuged as a batch process in a COBE 2991 machine to separate the different tissues. The gradient solutions themselves have also been shown to contribute to islet damage and may promote apoptosis [27, 28, 33]. This separation process can be difficult to control (especially with human preparations) because of intrinsic inconsistency in exocrine tissue density and variability in the extent of tissue digestion [25, 34, 35].

Several alternative approaches to density gradient centrifugation for islet purification have been explored, but none has proven to be a suitable replacement [25] despite the clear need for improvements. This can be partially attributed to limitations in scale and a lack of large throughput capability that is required for therapeutic islet transplant. Magnetic separation is one suggested method for islet purification and could offer significant benefits when compared to other methods. Magnetic separation techniques have been used to purify islets by selective magnetic labeling of the endocrine [34, 36] or the exocrine tissue [37]. Islet yields and transplant outcomes in rats were improved when magnetic microparticles (MMPs) were preferentially entrapped within the tortuous islet microvasculature and purified using magnetic retraction [38]. Furthermore, developments in quadrupole magnetic separation (QMS) technology have shown promise for the large scale batch purification of porcine islets [39], with no observed detrimental effects on islet function due to magnetic forces or shear stresses [15, 40, 41]. Suszynski et al. also demonstrate that MPs are well tolerated* in vivo*, with no observed increase in islet cytotoxicity or inflammatory responses in mice [42].

To further investigate the potential to improve isolated islet quality and viability, this study directly compares density gradient purification and QMS technologies. Three split-lobe porcine islet isolations were conducted to evaluate potential benefits of QMS. There is a growing interest in porcine islet xenotransplantation as a promising and cost-effective approach to alleviate the donor shortage [43, 44]. In addition, porcine donors are a good surrogate model for these purification comparisons due to the similarity in pancreas size to the humans and the consistency of donor parameters, procurement methods, and isolation parameters. For this study, QMS technology was further adapted for the continuous purification of porcine islets to reduce exposure to damaging digestion components and eliminate exposure to harmful density gradients. Islet quality was determined by morphology score and viability was determined by oxygen consumption rate normalized to DNA (OCR/DNA) [9, 45, 46], a predictor of islet transplant outcome [45]. Improving islet quality and viability will improve the potency of transplanted islet preparations and may significantly improve the success of single donor islet transplantation.

## 2. Methods

### 2.1. Experimental Design

This study compares the quality and viability of isolated porcine islets purified by either QMS or continuous density gradients (DGs). To control for donor variability and allow for paired comparisons between techniques, QMS and DG were performed on islets from separate lobes of the same pancreas which were digested using identical parameters. The pancreata were divided into two parts, with the first part being composed of the combined connecting and duodenal lobes (CDL) and the second part being composed of the splenic lobe (SPL) [47]. Islets in the SPL were labeled with magnetic microbeads as described below, and islets in the CDL were not labeled. Both lobes were digested simultaneously but in separate digestion chambers and using identical isolation parameters and materials to ensure identical warm and cold ischemia and similar digestion outcomes. Islets from the CDL were purified using standard DG purification, and labeled islets from the SPL were purified using an islet QMS system. Islet quality, based on morphology score, and viability, based on oxygen consumption rate normalized to DNA, were compared between conditions.

### 2.2. Donors, Procurement, and Labeling

All procedures involving animals were approved by the Institutional Animal Care and Use Committee (IACUC) and performed at the University of Minnesota. Three adult landrace porcine donors were used for this brief study, and all donors had similar mass (257 ± 3 Kg) and age (36 ± 0 months). Procurements were performed using previously described methods [47–49]. In brief, porcine donors were chemically sedated with a 100–500 mg dose of telazol, heparinized, and then euthanized with a fatal dose of sodium pentobarbital. Following confirmation of death, donors were exsanguinated and eviscerated, and pancreata were dissected from the viscera* en bloc *on the back table. During dissection, the aorta was located and the celiac trunk (CT) and superior mesenteric artery (SMA) were simultaneously flushed with 3–5 liters of cold organ preservation solution (CPS), and the pancreatic duct was infused with 60 mL of ductal preservation solution. The pancreas was additionally cooled by applying crushed frozen lactated Ringer's solution and irrigation. All pancreata experienced less than 20 minutes of warm ischemia. After complete resection with the vasculature intact, the pancreas was split into the CDL portion and the SPL portion. The native arterial vasculature was preserved with the SPL for infusion of MMPs. A previously optimized dose of 4.5 *μ*m-in-diameter MMPs (Dynabead M450, Invitrogen, Carlsbad, CA) was suspended in one liter of CPS (16 × 10^8^ MP/L) and infused into the splenic lobe through the CT and SMA using the hand syringe technique described by Rizzari et al. in 2010 [48]. After bead infusion, a second liter of CPS was flushed into the arteries to rinse out any beads that were not securely lodged in the microvessels. Both portions of the pancreas were then submerged in CPS and transported to the isolation facility in an ice filled cooler. All pancreata in this study experienced less than 250 minutes of cold ischemia before isolation.

### 2.3. Digestion

Islets were isolated from each lobe by the isolation team at the Schulze Diabetes Institute at the University of Minnesota in the same manner using the standard enzymatic digestion process [50, 51]. The new enzyme mixture described by Balamurugan et al. in 2012 for use with human islet isolation [52] was used to manually distend the pancreatic lobes from each pancreas. Following distension, the pancreas tissue was cut into 1 cm pieces and placed into a digestion chamber. Both the CDL and SPL from each pancreas were digested separately, using identical isolation parameters. The tissue from the CDLs was collected and purified by density gradients and tissues from the SPL were continuously purified using QMS.

### 2.4. Density Gradient Purification

Following the digestion phase, the digested tissue from the CDL of each pancreas was prepared for density gradient purification. The tissue was collected and recombined using the standard method in chilled conical tubes primed with heat inactivated porcine serum to reduce the enzyme activity. The tubes were gently centrifuged and the tissue pellets were collected into a single conical tube. The digested tissue was then separated by continuous density gradients (UW/OptiPrep Axis-Shield, Dundee, UK) using a COBE 2991 cell separator [51, 53] to attain a pure islet fraction.

### 2.5. QMS Purification

**Figure 1:**
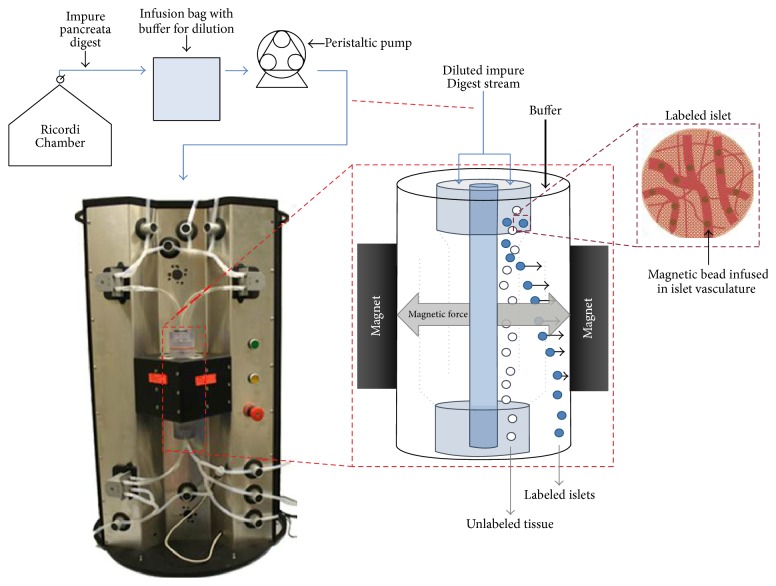
Schematic diagram of the QMS system used for the continuous purification of islets during digestion. The QMS replaces the collection and purification steps of the islet isolation process with a continuous QMS of labeled islets from the collection stream leaving the digestion chamber. This process streamlines islet preparation, avoids the harmful centrifugation, recombination, and gradient purification steps, and immediately washes and collects the purified islet product. Illustration by Chan A. Huynh.

After completion of the digestion phase, and when periodic sampling indicates that the islets are free, tissue from the SPL of each pancreas was separated by QMS. Rather than collection into conical tubes, the digested tissue containing MP labeled islets and unlabeled exocrine tissue was directly processed by the QMS system shown in Figure 1. Before the purification process began, the Islet QMS separator (Techshot, Inc., Greenville IN) [40] was primed with Hanks' balanced salt solution (HBSS) containing 10% heat inactivated porcine serum, and an infusion bag for flow buffering was prepared containing the same solution. The digested tissue left the chamber and was directly transferred to the buffer bag. The tissue only remained in the buffer bag for a few moments as it was quickly passed through the QMS system. The QMS inlet flow rate (out of the buffer bag) was matched with the flow rate of tissue leaving the digest chamber to maintain a continuous purification process. The mechanism for the QMS process using this system is described in detail by Kennedy et al. and others [15, 40, 54]. Briefly, as the tissue flow stream entered the QMS separator column, it is met with a parallel fresh stream of HBSS solution. Labeled islets are pulled by the magnetic force within the column out of the isolation stream and into the fresh solution stream and then remain within the column. Unlabeled exocrine tissue passes freely through the column and into a waste collection vessel. After all of the tissue has been processed, the column is removed from the magnet, and the purified islet tissue is gently washed out.

### 2.6. Isolation Outcomes and Islet Quality Assessment

After the purified islet fractions were collected from either the COBE bag or the QMS column, the islets were gently washed with fresh culture medium (supplemented ME199, Mediatech Herndon, VA). Samples were immediately assessed for quality by comparing islet morphology score [51, 55–57] and for viability by measuring the OCR/DNA as described in literature [45]. These methods are established and published and are only described briefly below. Qualitative morphological assessment was performed by experienced isolation personnel, where two samples of islets from each preparation were viewed under magnification and scored from 0 to 2 (each) based on the shape, border, integrity, diameter, and presence of single cells for a total score from 0 to 10 for each sample. Islets with better gross morphology have a higher score. Islet viability was determined by measuring the OCR/DNA using a titanium stirred microchamber system [46], and DNA was quantified using the Quant-iT PicoGreen dsDNA kit (Molecular Probes, Eugene, OR).

### 2.7. Statistical Methods

Small samples sizes are a limitation of this proof-of-concept study; nonetheless, average values are reported as the mean ± the standard error of the mean. The two-tailed paired Student's *t*-test was used to compare OCR/DNA measurements, while the nonparametric Mann-Whitney rank-sum test was used to compare islet morphology scores between groups.

## 3. Results

To explore the potential of using QMS to improve isolated islet quality and viability, this study was conducted in a pairwise fashion to directly compare the QMS purification method to the traditional method of DG purification using islets obtained from the same pancreata. Three porcine pancreata were split into two parts, with the first part (CDL) being isolated and purified by DG and the second part (SPL) being isolated and purified by continuous islet QMS. Isolated islets were compared for quality as determined by islet morphology score and for viability as determined by OCR/DNA measurements immediately following isolation.

**Figure 2:**
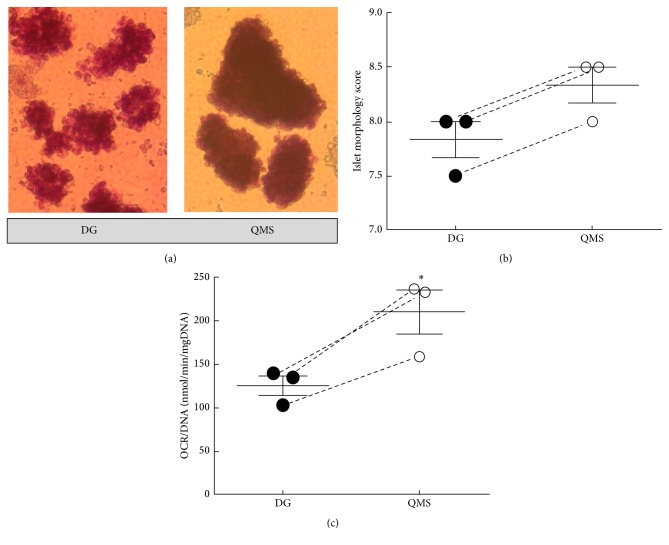
QMS improves islet morphology and viability. (a) Representative magnified images of islets stained with dithizone immediately following isolation purified using (left) density gradients (DGs) or (right) quadrupole magnetic separation (QMS). At higher magnification, it is easy to appreciate the improved gross morphology of islets purified by QMS. Islets purified using QMS noticeably exhibit a more robust appearance, with larger sizes, more defined borders, and less free single cells. Islets purified using DGs frequently have a more fragmented appearance, with smaller size and rough borders. (b) Islet morphology scores determined immediately following isolation and purification. Islets purified by quadrupole magnetic separation (QMS) show improved morphology scores compared to traditional density gradient purified islets. Higher scores indicate better islet shape, borders, integrity, and diameter along with reduced presence of single cells. The sample size is small, so the difference is not significant; however, QMS purified islets exhibited a better score for all three paired islet isolations. (c) Graph presenting the measured viability of islets purified using density gradients (DGs) or quadrupole magnetic separation (QMS) immediately following isolation. QMS purified islets have a significantly higher viability (*∗* indicates *p* = 0.03) than DG purified islets immediately after isolation.

Representative micrographs of islets from each condition are shown in Figure 2(a). Due to the small sample sizes used in this study, the difference in average islet scores is not statistically significant (*p* = 0.16), but the differences in gross islet morphology observed in the micrographs can be easily appreciated at the magnification shown.  Figure 2(b) shows a plot of average islet morphology scores of islets isolated and then purified using the DG and QMS systems. Islets purified by QMS had an improved average islet score when compared to islets purified using a standard DG system.

Oxygen consumption rate measurements were taken from samples of islets of each condition to determine the viability. Samples were collected and assessed immediately following isolation. The OCR/DNA of islets purified by QMS was significantly higher compared to DG purified islets immediately following isolation with mean values ± standard error of the mean (SEM) of 209 ± 25 and 125 ± 11 nmol/min/mg DNA, respectively (*p* < 0.03).  Figure 2(c) shows a 67% improvement in islet viability immediately following isolation. Comparisons of additional outcomes were beyond the scope of this proof-of-concept study due to small sample sizes and because sort parameters were not optimized for this purpose.

The number of islets transplanted per recipient kilogram body weight (IE-Tx/kg BW) has proven to be predictive of clinical transplant outcome in the human autograft and allograft model [2, 6, 58–60]. Combining this with the islet oxygen consumption rate to calculate the total OCR transplanted per kilogram body weight yields an even more reliable benchmark for predicting insulin independence in auto- and alloislet transplantation [45, 61–63], and this metric encompasses both the total amount of tissue transplanted and the viability of that tissue. By applying this approach to previously presented data [62, 63], the viability improvements observed with QMS would improve insulin independence rates from 29% to 69% in autograft patients and from 38% to 92% for allograft recipients.

## 4. Discussion

Islet allotransplantation is a promising therapy for the treatment of type 1 diabetes, and islet xenotransplantation offers the opportunity to alleviate donor shortages and expand the application of this treatment to a larger patient population. Islet transplantation offers many benefits over other therapies such as whole pancreas transplantation or insulin therapy [64, 65]. Despite these benefits, one of the primary barriers to expanding the application of islet transplantation is the frequent need for multiple islet transplants, from 2 to 3 donors, to maintain insulin independence, often in quick succession. This requirement puts further strain on the already stressed organ donation programs and prevents the growth of this promising treatment. Some centers have encountered success with single-donor transplants [6, 7], but most centers still need multiple donor pancreata to meet the very large total islet dose required for patients to remain insulin independent [3–5, 64]. Experience in pancreatic surgery suggests that patients can avoid diabetes even when large portions of their pancreas are removed, and research suggests that patients do not become diabetic until a large portion of their islet function fails [66–68]. These experiences suggest that current islet transplant dose requirements have significant room for improvement. There are many factors that contribute to this large dose requirement, but the potency of transplanted islets and their function in the body after transplant are of paramount concern. The potency of an islet preparation is affected by a plethora of factors including donor health, extended exposure to brain death, pancreas condition, organ procurement and preservation methods, and islet isolation and culture methods. This work focuses on improving islet quality and viability which directly affects the therapeutic potency of the islet transplant and provides islets the best chance of engraftment and survival.

Much attention in the field is given to improving the enzymatic digestion step of the isolation process, but there is also significant damage to islets during the purification step [25–28]. Islets experience very harsh conditions that inflict lasting damage during the mechanical and enzymatic digestion process [19, 20], but the current DG purification method significantly prolongs and exacerbates this damage. This study explored the use of QMS technology as an alternative method for the purification of liberated islets to significantly improve islet quality and viability. Porcine islet morphology as reported by the islet score can be a gross indicator of damage that occurs during the digestion and purification process. Islets from porcine donors do not have a robust peri-islet capsule that is often observed surrounding human islets, and this can make them more sensitive to damage associated with the isolation process. Frequently, islets are observed with rough and uneven borders indicating damage to the cells around the islet periphery. The results presented clearly demonstrate that QMS purified islets have improved morphology and significantly improved viability immediately following the isolation process.

These improvements can be attributed to eliminating the use of harmful DG chemicals [27, 28, 33] and the numerous functional benefits of QMS over the traditional DG purification method. During the digestion process, when the tissue within the closed circuit is considered to be adequately digested so that most or all of the islets observed in periodic samples appear to be free from the exocrine tissue, the digestion phase ends, and the collection phase begins. This is commonly called the “switch” point, when closed loop circuit is opened, and the digested tissue is slowly collected. Tissue that is not completely digested remains within the chamber to continue the digestion process. Perform purification using DG methods then expose islets to extended periods (up to an hour) of warm and cold hypoxia in the digestion solution during the collection, centrifugation (recombination), and COBE processes. When using the continuous islet QMS system, these steps are completely eliminated, and the digested tissue is transferred directly to the QMS system, where the islets are almost immediately processed and removed from the harmful digestion solution.

The islet QMS system is a continuous sorting technology previously described in the literature [15, 40], where the stream of digested tissue travels through a uniquely designed separation column that is surrounded by a powerful rare-earth magnet. When the tissue stream enters the column, it is met by a cool fresh solution stream that travels through the column along with the tissue stream in a laminar flow, so that the two streams have minimal mixing. Islets that are labeled with magnetic beads are quickly but gently “pulled,” by the magnetic forces, out of the digestion stream and into the fresh solution stream. The digestion solution contains many of the harmful enzymes, cytokines, and reactive oxygen species that can damage islets. Using the continuous QMS method within a minute or two after leaving the digestion chamber, the islets are removed from this damaging environment and washed with a fresh solution stream. For these studies, which used nonoptimal QMS technical parameters (e.g., flow rates), the separated islets were pulled to the sidewall of the column and remained within the column for the duration of the purification. During this time, the purified islets were continuously washed with a stream of fresh chilled solution. After the purification process was complete (shortly after completion of the digestion process), the column was sealed and carefully removed from the QMS system, so that the purified islets could be washed out and collected for culture and quality assessment.

In this study, QMS purified islet viability as measured by OCR/DNA immediately following isolation showed a 67% improvement over DG purified islets. For cases like autoislet transplantation, when islets are almost immediately infused following isolation, these results could have profound implications. Autoislet transplants are routinely done for patients who suffer from chronic pain associated with pancreatitis. The pancreatectomy is done to alleviate pain, and the autogeneic islets are infused to avoid diabetes. Often, the pancreas in these patients is in poor condition with calcifications and fibrosis, which can significantly reduce the islet yield obtained during isolation. This makes the maintenance of islet dose and potency primary concerns when total islet mass is limited. To ensure a maximum amount of transplanted islets, sometimes the purification step is avoided and the total amount of unpurified tissue is infused. Literature suggests that this practice may be detrimental to islet engraftment and function [21–23] due to the large presence of digested exocrine tissue. Some centers will do a purification step using modified density gradients to decrease the amount of exocrine tissue and total tissue volume for transplant. In these cases, when purification is desired, using QMS instead of DG could have a substantial impact on graft function because the purified islets would have increased viability and a better chance of survival after transplant. For autograft recipients, more than twofold improvement in insulin independence rates is predicted for islets purified by QMS. More studies which focused on the application and optimization of QMS as a potential method for purification of islets for autotransplant cases are of critical importance.

QMS could have an even greater impact on islet allotransplantation outcomes, because islets are almost always purified using DG methods and are frequently cultured for 1-2 days following isolation. With significant improvements in viability, the islets will survive better in culture, and this will increase the total number of viable islets available at the time of transplant. A 92% insulin independence rate is predicted for islet allografts if islet viability was improved by 67% as observed with QMS purified islets. Future studies comparing the postculture recovery of human islets purified by QMS and DG would be extremely valuable and would illuminate the potential of this method for use in allotransplant applications and may improve the consistency of purification which is problematic at present. QMS purification methods could be a big step towards the success of single-donor islet transplantation.

Furthermore, as the application of porcine islet xenotransplantation holds much promise for alleviating the donor organ shortage, continuous islet QMS could replace DG purification methods and increase the consistency and viability of isolated islet products. The field of islet xenotransplantation faces many challenges and islet potency has been determined to be a critical metric for approval of this cellular therapy. Xenogeneic islets will be subject to great scrutiny and will require extensive characterization and quality control. QMS can eliminate the use of damaging DG chemicals, simplify and streamline the purification process, and improve overall preparation quality and potency.

The results of this proof-of-concept study demonstrate that QMS is a promising purification method for improving isolated islet quality and viability. Numerous technical parameters (e.g., flow rates) are associated with the QMS system and critical studies with larger scope are required to optimize the process for improving yield and purification efficiency before clinical implementation. Further improvements and adjustments in equipment design and implementation for specific applications (e.g., xeno-, allo-, or auto-transplants) may be required and may expand the benefits observed in this early study. It should also be noted that the islet labeling methods used for this study were established in literature [38, 48], in conjunction with QMS equipment and process improvements. Additional investigation is required to enhance infusion and labeling for human and porcine islets.

## 5. Conclusions

To conclude, this study establishes that QMS technology adapted for islet separation can be used to significantly improve islet quality and viability immediately after isolation as compared to DG purification. Despite the minimal data set, the results presented suggest that, with further optimization, QMS could be a superior method for islet purification. This technology offers great promise for improving the viability of isolated porcine islets, and this application is becoming more relevant as xenotransplantation is approaching clinical application. QMS technology has the potential for a more immediate application to improve clinical outcomes for select islet autotransplantation cases and nearly all islet allotransplantation cases by eliminating the use of DGs and improving overall islet preparation viability. There are many challenges to overcome for the large scale implantation of islet transplantation for the treatment of type 1 diabetes, but improving isolated islet viability is a major step towards single-donor success in allotransplantation and implementation of porcine islet products for xenotransplantation.
